# Decision making process and factors contributing to research participation among general practitioners: A grounded theory study

**DOI:** 10.1371/journal.pone.0196379

**Published:** 2018-04-25

**Authors:** Seng Fah Tong, Chirk Jenn Ng, Verna Kar Mun Lee, Ping Yein Lee, Irmi Zarina Ismail, Ee Ming Khoo, Noor Azizah Tahir, Iliza Idris, Mastura Ismail, Adina Abdullah

**Affiliations:** 1 Department of Family Medicine, Faculty of Medicine, Universiti Kebangsaan Malaysia, Kuala Lumpur, Malaysia; 2 Department of Primary Care Medicine, University of Malaya Primary Care Research Group, Faculty of Medicine, University of Malaya, Kuala Lumpur, Malaysia; 3 Department of Family Medicine, School of Medicine, International Medical University, Seremban, Malaysia; 4 Department of Family Medicine, Faculty of Medicine and Health Sciences, Universiti Putra Malaysia, Serdang, Malaysia; 5 Cyberjaya University College of Medical Science, Cyberjaya, Malaysia; 6 Klinik Kesihatan Ampangan, Seremban, Malaysia; 7 Klinik Kesihatan Seremban 2, Seremban, Malaysia; University of Göttingen, GERMANY

## Abstract

**Introduction:**

The participation of general practitioners (GPs) in primary care research is variable and often poor. We aimed to develop a substantive and empirical theoretical framework to explain GPs’ decision-making process to participate in research.

**Methods:**

We used the grounded theory approach to construct a substantive theory to explain the decision-making process of GPs to participate in research activities. Five in-depth interviews and four focus group discussions were conducted among 21 GPs. Purposeful sampling followed by theoretical sampling were used to attempt saturation of the core category. Data were collected using semi-structured open-ended questions. Interviews were recorded, transcribed verbatim and checked prior to analysis. Open line-by-line coding followed by focus coding were used to arrive at a substantive theory. Memoing was used to help bring concepts to higher abstract levels.

**Results:**

The GPs’ decision to participate in research was attributed to their inner drive and appreciation for primary care research and their confidence in managing their social and research environments. The drive and appreciation for research motivated the GPs to undergo research training to enhance their research knowledge, skills and confidence. However, the critical step in the GPs’ decision to participate in research was their ability to align their research agenda with priorities in their social environment, which included personal life goals, clinical practice and organisational culture. Perceived support for research, such as funding and technical expertise, facilitated the GPs’ participation in research. In addition, prior experiences participating in research also influenced the GPs’ confidence in taking part in future research.

**Conclusions:**

The key to GPs deciding to participate in research is whether the research agenda aligns with the priorities in their social environment. Therefore, research training is important, but should be included in further measures and should comply with GPs’ social environments and research support.

## Introduction

In many parts of the world, networks and support have been established to facilitate research activities in primary care [1–4]. Increasingly, studies are conducted based on active participation of general practitioners (GPs). Active participation in research is a crucial aspect in these networks and in conducting primary care studies [5,6]. However, literature from some countries still shows low levels of GP participation in research activities [7–10]. In Malaysia, the Malaysian Primary Care Research Group (MPCRG) was established in 2004 to encourage primary care research and to build research capacities among practicing GPs. Although MPCRG membership increased to about 250 in 2016, less than 10% of members actively participate in research [11]. Primary care research output in Malaysia is mainly driven by primary-care academics.

The reasons for such poor participation have been explored, and include time and staff constraints, irrelevant research topics and poor remuneration and rewards from research activities [10, 12–19]. Practicing GPs may view participation in research activities as a daunting task. Nevertheless, there are also motivating factors for research participation, such as the availability of a mentoring program, access to research information, relevant topics, opportunities to participate and personal interest in research activities [9,16,20]. Although a few supporting or preventive factors have been identified, there is still no convincing explanation why GPs decide to participate in research studies. An understanding of the decision-making process to participate in research would necessitate an understanding of the interactions between a GP’s personal factors, their practice and the health system to facilitate the identification of opportunities for improving research participation. Therefore, we aimed to develop a substantive and empirical theoretical framework to explain GPs’ decision-making process to participate in research.

## Methods

We adopted grounded theory methods for this study as they provide systematic steps in the construction of a theoretical framework [21] that aid the description of the process one undertakes to arrive at certain actions [21–23].

### Study setting and participant recruitment

The study was conducted in primary care settings in Malaysia. Primary care services in Malaysia are provided by dual public-funded and private fee-for-service clinic systems [24]. Each system has different means of remuneration, which could affect a doctor’s decision to participate in research. Thus, at the initial phase of the study, we selected 15 GPs with varying experience in research participation from both public and private clinics. One GP had never participated in any research activity, one had conducted research in the past but had ceased to do so in the past few years, three had just embarked on projects due to course requirements, six had participated in data collection and four were leading projects. Four GPs participated in in-depth interviews (IDIs) and the remaining 11 participated in one of three focus group discussions (FGDs). After initial analysis, a further 11 GPs, 10 from public and one from private clinics, were recruited to participate in a further two FGDs and one IDI. These GPs were selected based on their current career stage, as we learnt that life stages have a significant impact on the decision to participate in research. All GPs were recruited through the personal contacts of MPCRG committee members.

### Data collection

We selected IDI as one of the data collection methods because it allows participants to express opinions in a safe environment [25]. Non-participation in research might be perceived negatively, i.e. as undesirable. Prior experience in research participation can also be regarded as personal information that might not be shared in FGDs. On the other hand, FGDs encourage discussion of the challenges participants might face in participating in research, which we presumed would be issues shared by the majority. The participants were briefed on the study objectives, and confidentiality was assured. The interviewers or facilitators introduced themselves as university colleagues who wished to learn from the GPs’ opinions and experiences in research participation. Participant information sheets were issued, and any doubts were clarified before consent to participate was obtained. The consent included permission for audio recording and transcription. All interviews were audio-recorded and transcribed verbatim prior to analysis. The interviewers and fellow researchers checked each transcript for accuracy of transcription.

### Question guide

The initial questions asked were general questions with the aim of inquiring about the participants’ experience with research (Box 1). This was consistent with the concept of being open to all possibilities in the construction of the substantive theory.

**Box 1.**

1. What is your opinion of evidence-based medicine and research in general practice?
2. Have you had any experience carrying out research activities in your clinic?
3. How did you decide to participate in research?
4. Can you describe your experience? How were you involved? Who did you collaborate with?
5. What are your thoughts and feelings about the experience?
6. What were the difficulties you faced?
7. If you have not had the experience, what are your thoughts on carrying out a research project in your clinic?
8. How do you manage research activities with your daily routine in the clinic?
9. Has your participation in research been useful to your practice?
10. What value do you see in research in primary care?
11. Would you embark on any research activities in the future? What are your reasons?

The questions served as a guide, and participants were encouraged to elaborate as much as possible. After initial analysis of a FGD and five IDIs, particularly after re-analysing the response from the fourth IDI, we included questions exploring the participants’ experience in striking a balance in research, clinical work and personal life because we learnt that the doctors’ personal life or life stages were integral to their decision to participate in research. These questions were added in the subsequent (fifth) IDI and four other FGDs. We also included questions on the GPs’ early exposure to research, as this was noted as one of the leading factors for participation in research activities. During debriefing at the end of all sessions, the participants were asked to comment on any issues that had not been raised or any concerns they had during the sessions.

### Analysis

The data interpretation was based on symbolic interactionism. Symbolic interactionism assumes that people act on an object based on the meaning they assign to it. The meaning is constructed through a person’s social interactions with others [26]. Therefore, actions are the focus of interpretation in constructing a theoretical framework [21, 22]. Using this theoretical underpinning, we focused on the GPs’ actions and decisions leading to participation or non-participation in research activities. Nevertheless, it was difficult to observe the actions taken by the participants and their decision-making process. Instead, we attempted to reconstruct their actions and decision-making process based on their descriptions of their experiences. Focusing on this reconstructed action would also facilitate study of the relationship between various concepts, i.e. barriers, motivators, factors and needs, in the complete process of research participation.

The initial analysis involved line-by-line coding, and gerunds were used as much as possible to allow us to reconstruct their actions [21. 22]. The ‘action’ codes facilitated the development of the theoretical framework [22]. The codes were subsequently reviewed, discussed and grouped to construct a higher level of abstract codes—the categories [21]. Focus coding ensued, when categories were sufficient to significantly capture the meaning of the data, and the categories were used as a template to analyse the remaining transcripts. Theoretical coding is the final step in constructing a theoretical model. The research team discussed and hypothesized on the relationships between the categories to link the fragmented categories into a coherent theoretical model [22]. During the first round of analysis, SFT coded four transcripts (two IDIs and two FGDs). The codes and categories were discussed among the research team members, who had read the transcripts prior to meetings. The research team provided a bird’s eye view of the transcripts to complement interpretation from the detailed line-by-line coding. Some categories, i.e. ‘Fitting into life stages’ and ‘fitting into the profession’, were constructed from the meetings. These categories were used to inform further data collection and coding. The coding and discussion cycles continued until theoretical saturation was achieved. Further data collection and analysis did not add significantly to our understanding of GPs’ participation in research. The codes and categories were defined and refined throughout the analysis. A core category of ‘fitting research agenda to social environment’ was chosen as the key to research participation. The whole analysis process was facilitated by constant comparisons between codes, categories and data. Memos were also used to capture ideas and interpretations of the data during the analysis.

### Ethical consideration

We sought consent for participation and audio tape-recording prior to each IDI or FGD. We explained the objective, purpose and process of the interviews to the participants, and assured them of the confidentiality of the interview contents and their identities. Transcripts were anonymized prior to analysis. The audio files were erased after the transcripts had been checked for accuracy. Each participant was compensated for their time spent in the interviews or focus groups. Interviews were scheduled at the participants’ convenience. Ethics approval was obtained from the Universiti Kebangsaan Malaysia Medical Research Ethics Board (FF 313–2012) and the Malaysian Ministry of Health (NMRR-12-894-13579).

## Results

A total of five IDIs and five FGDs were conducted; 26 doctors participated in the study. Table 1 provides a summary of the participants’ backgrounds. We recruited GPs from public and private sectors of various ages, research experience and training. FGD 1 consisted of participants who were post-graduate trainees in general practice, where research was one of the course components. FGD 3 consisted of participants with no formal research training and experience. One participant in IDI 2 had no formal research training but had been actively leading research projects in collaboration with other experienced researchers. This participant worked in the private sector, where there is very little support to conduct research.

**Table 1:** The participants’ background characteristics.

	Focus Group Discussion	In-depth Interviews
Number of participants	21 (5 FGDs)	5
Private sector	11	5
Public sector	10	0
Age range (years, maximum and minimum)	25–69	32–55
Research experience		
Yes	11	3
No	10	2
Research training		
Yes	10	1
No	11	4

**Fig 1:**
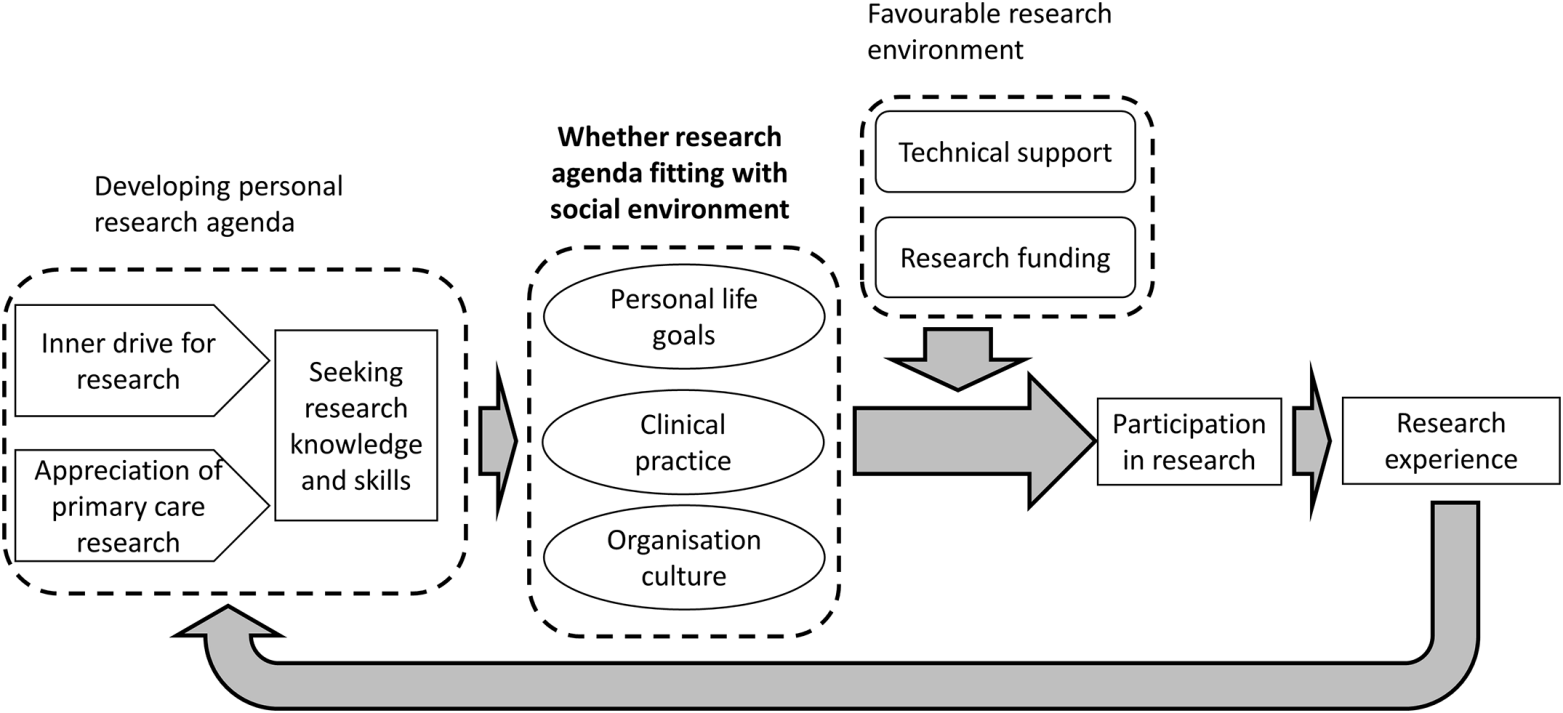
Factors and decision-making process leading to GPs’ participation in research.

Fig 1 shows the factors and decision-making process leading to the GPs’ participation in research. Three main factors contributed to the decision to participate in research: 1) personal research agenda, 2) the social environment, and 3) favourability of the research environment.

However, the key to GPs deciding to participate in research was whether the personal research agenda fit with their social environment. A positive personal agenda for research and good research knowledge and skill were insufficient for the GPs to decide to participate in research. A supportive research environment would facilitate their involvement in research only if the research agenda fit with their social environment. Although the decision-making process of research participation in Fig 1 appears to lead from one stage to another, all three factors operated simultaneously. The flow merely serves to facilitate understanding of the decision-making process.

### Developing a personal research agenda

Personal research agenda refers to the GPs’ personal views and interest in research. The research agenda also encompasses their stance on evidence-based medicine (EBM). This was developed from their inner drive to do research, and over time, the value the GPs placed on primary care research (Fig 1). Some GPs perceived research as a professional obligation and personal quest for knowledge; this formed their inner drive to perform research. This had an element of inbuilt conscience and enthusiasm. The enthusiasm ranged from personal interest to seeing a need to contribute to the discipline of general practice.

*So to me research is a joy… ok, they’ve always always been in my mind… It is aah it’s on our conscience what we want to do, you see*.

- *55 years old, private practice (IDI)*.

*Since I’m evidence user, of course I do have the feel the need to contribute in research in terms of developing a good health system*.

- *44 years old, private practice (FGD)*.

In contrast to inner drive, appreciation for primary care research implied an acquired characteristic from training or working as a GP. Previous research experience during GP training and exposure to research promoted the GPs’ interest in primary care research. They had encountered teachers who inspired them to perform research or had found existing evidence not applicable to informing their primary care practices.

*Yes, because you know this master programme [in family medicine], we need to do this thesis, study and … then after graduation, then I’m thinking why not just go on [to do another project]*.

- *44 years old, private practice (IDI)*.

*Because all the data, all the research that you have is not at a primary care level, it’s at a secondary care level. So if you want your research findings to be applicable to a primary care setup, then you have to do it at a primary care level*.

- *55 years old, private practice (FGD)*.

Two participants spoke about how they became more interested in research after becoming involved in research activities during their careers as GPs. They felt enlightened by their involvement in research. They appreciated research in the primary care setting.

*I mean the reviewers although they didn't reject our articles there were lots of mistake on the way, you know. So I think that’s a journey that I never regret because I learned*.

- *55 years old, private practice (IDI)*.

*The process of the research itself like I mentioned earlier perhaps it’s very—it’s very reflective in—in—in enlightening you to realize your own practices, your own prejudices and things you have done*.

- *48 years old, private practice (FGD)*.

On the other hand, a lack of exposure to understanding primary care research, not embracing the values of EBM or failure to see a need for further research could put the GPs off research.

*Maybe because of my exposure to this type of research [research in primary care setting] is very limited, that’s why I couldn’t really see what [research] can be done in this type of setting*.

- *47 years old, private practice (IDI)*.

*Or me even EBM also they [patient] may not even bother so—so for them there’s—there’s no point of doing research*.

- *44 years old, private practice (FGD)*.

Having an interest and appreciation for research may not necessarily lead to the GPs engaging in primary care research, as they need to consider many other challenges; however, not having an interest and appreciation for primary care research at all would certainly stop GPs from considering participation in research.

### Fitting with the social environment

The key to determining the degree of GP participation in research was whether such participation fit with the GPs’ social environments. As a member of society, the degree of their participation in research depends on how they manage their various responsibilities. Full-time GPs perceived research as an added responsibility and not part of their core activities. Each participant had differing responsibilities and priorities, including personal goals and life priorities related to family, clinical practice and organizational culture. The degree of participation in research would demand a portion of their resources (either in terms of time or material cost) being removed from their social life. Thus, if a GP was successful in striking a balance between research activities and social life, he would participate in research. These social responsibilities can be conceptualised into personal life goals, clinical practice and organizational culture.

#### Personal life goals

Personal life goals change over time. Junior doctors would want to concentrate on building their career and family, while the more established and senior doctors would not mind balancing their life goals to include research activities. Nevertheless, a research-motivated junior doctor would participate in research activities if they have established a balance to fit research work into their social life. Thus, the key is to establish a balance between their personal research agenda and life goals.

*It really also depends at—at what stage in life or at what stage in practice you are in. If you were talk—you were to talk to me 10 years ago, I would shut you off straight away. I wouldn’t have time*.

- *44 years old, private practice (FGD)*.

Personal goals varied between individuals, and included allocating time and resources for leisure, daily chores, family and personal financial needs. Research was often seen as financially unrewarding. Striking a balance between life goals and research was seen as a strain on their personal time and resources.

*I like to study and I like to read but in reality when we have a—we have—we have our own life and then we have our—our family time*.

- *32 years old, private practice (IDI)*.

#### Clinical practice

Research must be of interest and be able to fit into a GP’s clinical practice. It must be feasible in order to be undertaken. Research participation must be seen as relevant to clinical practice, not cause undue disruption to the clinic routine and not adversely affect the practice revenue. The GPs also needed to consider whether their patients would accept invitations to be participants in research projects. The latter was a significant factor in the private sector, where patients pay for health care.

*I mean if given the right time and I mean the right research that was my interest, I think I would commit to do the research*.

- *30 years old, public practice (FGD)*.

Significant disruption of the clinic routine and time constraints were discussed in almost every interview and focus group. The key was the ability to adjust the time and fit research activities into the clinic routine. Participants spoke about ‘making time’ in the context of an issue of priority rather than time constraints per se. Thus, simple research protocols are more likely to be implemented than complex research designs. Nevertheless, the GPs would adjust their routines accordingly if they had strong interest in the research.

*I just couldn’t find time or maybe I don’t make time to do research*.

- *52 years old, public practice (FGD)*.

*But of course I was very willing [to be involved]. I thought the idea was good. But when I looked at the implementation and I thought it was… difficult. Huge [amount of work]*.

- *44 years old, private practice (FGD)*.

*I’m not seeing a lot of patients, so it’ll be a good place I think to do research*.

- *37 years old, private practice (IDI)*.

Among the private-sector GPs, practice revenue was a factor in deciding to participate in research. Generating sufficient revenue took precedence over research participation, albeit the degree of sufficient revenue was subjective. GPs have different financial needs throughout their life stages. In a more established practice with a stable practice list, doctors may consider participating in research when less burdened by finances as compared to younger practitioners.

*Less-established GP, their main concern is still finance. They just want to survive. Imposing research responsibilities on them is maybe quite a bit something unless there is a definitely financial gain. Yeah. Because time is—our time is money*.

- *48 years old, private practice (FGD)*.

Engaging in research was seen as an encroachment on a GP’s practice, revenue and family life. Therefore, the GPs were more likely to participate in research activities if there were monetary incentives.

*Besides taking care of patients, we have to take care our business, our clinic, we have to take care our family, and here you asking us to fill up diaries and fill up this. It’s—it’s—it’s extra burden*!…

- *44 years old, private practice (FGD)*.

…*unless there’s a benefit that the clinic can get, maybe reduce the tax*.

- *32 years old, private practice (IDI)*.

Nevertheless, achieving a balance between clinic revenue and research participation would depend on a GP’s agenda for research. The participant in IDI 2 had a strong interest in research, thus he devised a feasible financing method that balanced clinic revenue and research project funding.

*The patients pay part of it [price for full investigation], I pay part of it. Because, for example, [patient pay the urine culture], the cost price, maybe about 15 ringgit (Malaysian currency). We [usually] charge [non-research] patients probably 35 ringgit. So, I will be kind of financing them on a 20 ringgit*.

- *55 years old, private practice (IDI)*.

Perception of patients’ acceptance of research activities also influenced GP participation in research. The concern was whether their patients perceived that they were being taken advantage of in participating in research projects. To overcome this concern, a compensatory mechanism for patients was established in terms of their time spent to compensate for the inconvenience caused by the research project. This would encourage GP research participation.

*I feel that, you know, may be the patients will feel that we are making use of them*.

- *55 years old, private practice (IDI)*.

*We ensure that the patient that is we are going to subject this research we spend a little bit more time [explaining the study protocol] on them*.

- *55 years old, private practice (IDI)*.

*So, they—they actually gain from that as well. And they will be given a BP set, glucometer*.

- *50 years old, private practice (FGD)*.

Thus, issues within the practice setting must be addressed to accommodate research activities. Positive response to research participation was more likely if the research protocol was simple and with minimal disruption to clinic routines and revenue and if research activities were acceptable to patients. Otherwise, accommodating significant disruptions to clinic practices would require a GP to have strong research interest and appreciation for primary care research before he would agree to embark on a project.

#### Organisation culture

Besides practice settings, a research agenda must also fit into the larger organisational culture encompassing the practice. The issues of organisational culture encompass a reward system and recognition from performing research.

*That’s why in private GP, I think lack of motivation, recognition… like nobody care about you [doing research]… hahaha*.

- *44 years old, private practice (IDI)*.

To some GPs, establishing a niche among their peers was seen as a form of recognition. This tended to occur when a health system creates the opportunity for competition among practices. Research participation was seen as one of the potential niche areas they could tap.

*So in this continuing kind of competition you know we need to survive… so we need to look for new market. So that’s where you know we come to think of new things, so for example, aaa, in terms of the study which I did, which one of it is the on the diabetic retinopathy which I was thinking is that nobody has done it before*.

- *55 years old, private practice (IDI)*.

Researching and publishing can gain recognition from specialists and aid academicians in their advancement up the career ladder. Research is a requirement in advanced GP training programmes.

*But if you have written something which is up-to-date and evidence-based, the specialist actually respect you and wrote back if you are right*.

- *55 years old, private practice (IDI)*.

… *if I don’t do anything [in research], I’ll just be 54 [employment grade] until I retire. I thought, I must make an effort and I did*.

- *55 years old, public practice (FGD)*.

*It is this like ATP [a training program], there’s somebody want you to do [research]. So, you have to do*….

- *44 years old, private practice (IDI)*.

Thus, an organisational culture that has a reward system for research would encourage research participation from GPs. Rewards could take many forms, such as a promotion, career advancement or peer recognition.

### Favourable research environment

‘Research environment’ referred to resources, funding, facilities, manpower, technical support and research network to support research. Technical support included accessibility to methodological experts and availability of resources for literature search. These were considered important for facilitating research participation.

The researchers, the research place, the equipment, and then the training for the equipment as well, do you have the training before that, you know, can you access [to it?], how to do it, you know. Things like that. How much support do you have for the research!

- *37 years old, private practice (IDI)*.

The problem is in having the formative stage. That means… for us to finalise our problem statement. Most of the workshops are on methodology… on quality research. But I don’t see much workshops on… what is the appropriate way in framing the primary question?

- *50 years old, public practice (FGD)*.

The availability of technical assistance has to be coupled with favourable practice support for GPs to access technical assistance. GPs had to allocate time away from their clinical practice to access help.

CRC [Clinical Research Centre] has told us a couple of times, if you need any help, come over to CRC. But I don’t know… I never push myself hard enough. The time constraint!

- *52 years old, public practice (FGD)*.

Funding was a major issue. The GPs perceived it as less accessible and there was a lack of funding for primary care research.

So, if you’re going to [get] support proposal… you do face a problem of where to submit [for funding]. Don’t know whether they [Ministry of Health] will support or not. Because you know the allocation of budget is for them, right?

- *44 years old, private practice (IDI)*.

Networking was considered important to obtain technical support to facilitate research participation.

*When I was in the Ministry of Health last time, like any problem, I can consult this a statistician in public health, even academics also… but I’m doubtful because now I’m in private. I don’t know whether they have the impression… ‘Eh, why this private GP comes to us now?’ Because there’s no link you know… between Ministry of Health… and private GP*.

- *44 years old, private practice (IDI)*.

Peer influence for research could motivate and facilitate GPs to participate in research.

*Although there are things that we were behind [about research], but he [peer researcher] gave us the lead, the references, it was great help you know*.

- *55 years old, private practice (IDI)*.

*I had zero knowledge about research. I’ve never been interested in research. These two friends dragged me in*.

- *50 years old, private practice (FGD)*.

Despite their interest, the absence of research support would curtail the GPs’ enthusiasm for research.

*These [research ideas] are all thoughts that come to me [now]. But afterwards, there’s no one to talk to me today. But after you go back, I go to sleep (haha)*.

- *56 years old, public practice (FGD)*.

### Establishing priorities within the social environment

GPs had to decide between research, personal goals, family priorities, clinical practice and organizational culture, and it is difficult to accommodate all of these demands. GPs who were interested and wished to participate in research would have to place research participation on their list of priorities.

*Yeah. So you—you—most people like we would either choose—either choose clinical or you choose research or you choose aa financial, you know. Hahaha. It’s difficult to combine three to gain*.

- *37 years old, private practice (IDI)*.

*So to me research is a joy, I was telling thing that my friend would buy an expensive hi-fi, you know, but I, but for me I would probably spend that money on research*.

- *55 years old, private practice (IDI)*.

Life goals and priorities changed with time as a doctor moved from a junior to senior position. The GPs were active players in shaping their environment. Thus, with a strong personal research agenda and favourable research environment, they would create an opportunity within their social environment to accommodate their research activities. The effort in creating this opportunity was proportionate to the intensity of their desire to do research.

## Discussion

This study aimed to develop a theoretical framework to explain the decision-making process of GPs’ participation in research. The decision-making process involves interaction between the GPs’ research agendas and their social and research environments. However, the key step that underpins the decision is how GPs prioritise their research amongst other agendas in their social environment. Although GPs were bound by their personal life goals and clinical responsibilities when considering research, they would actively fit their research agenda into their social environment if they felt research was a priority. Their social environmental factors, be it personal life goals, practice and organisational matters, would then become secondary.

Whether the GPs prioritized research over other factors can be seen from how they allocated time for social activities, as shown in our framework (Fig 1). The time allocation underlines many commonly cited external barriers (or factors) to GP research participation, such as time constraints, poor remuneration, low rewards for research activities, staff constraints and complicated study protocols [10,12,14,16,17,19]. Remuneration and rewards are issues of whether the time spent on research is worth the effort compared to other activities in the social environment. If the GPs felt that the rewards are substantial, it would become a priority and they would decide to participate in research. The GPs would take the effort to invest and allocate time for research activities whenever possible, such as over lunch, during weekends, or reduce time spent seeing patients. Similarly, staff constraints and complicated study protocols would become barriers to the GPs’ involvement in research if they felt that research was not a priority compared with other activities in their social environment. The GPs’ allocation of time for research activities reflects their personal life priorities. Previous studies often did not highlight this, commonly citing only external barriers [10,12,14,16,17,19]. Personal life priorities differ from interest and attitude towards research, referring to the GPs’ life choices between family, clinic practice and research. Despite the interest in research, some may still prioritise clinic and family responsibilities. These priorities would change over time as the GPs advanced in their careers and seniority. They would choose to participate in research when the moment was right for them, for example at a later stage of life when they wanted to readjust their life priorities. They would fit their research agenda over other social priorities, whether it be personal or pertaining to clinic practice.

The other external factor influencing research participation is the health care structure [15]. Michalec et al. looked at interactions between GPs and the payment system in the American health system, a macro health care structure. In that study, GPs would participate in research if the research activities did not disrupt the *flow* of the clinic to attain practice outcomes, i.e. clinic revenue, patient health outcomes and clinic dynamics [15]. Many of these practice outcomes were beyond the control of the clinic or the GPs; rather, they were organisational-related, a macro structure in the health care system. We also noted that organisational culture and structure were factors in GPs deciding on whether to participate in research. Organisations that embrace research and provide a systematic structure to facilitate and encourage research would have a positive influence on research participation. Such organisations can be government bodies, large health care organisations or small organisations such as solo GP private practices. Although organisational culture and the GP could have mutual influence, we preferred to use the term ‘fitting in’ over ‘influence’ because, in our findings, the GPs played an active role in accommodating research activities over other agendas if the priority for research was high.

Community distrust of research is another external barrier to participation in research [14]. In the present study, the perception of patient acceptance of research activities in the clinic, which is part of the social environment, would partly influence the GPs’ decision to embark or participate in research activities.

Personal interest, relevant research topics and attitude to research also have a strong influence on GPs’ research participation [8, 10, 17,18]. In the present study, these were the main internal motivations for the GPs to actively fit research activities in their social environment. These motivations drove their interest or intensity in pursuing research and seeking research skills. We observed that some GPs expended extra effort to conduct research despite facing the same ‘barriers’ their colleagues did. We believe this is due to their strong interest and drive for research. This appreciation for research could change, which concurs with the literature stating that training and early exposure to research may alter GPs’ attitudes and interest towards research [27]. Nonetheless, we also recognise the importance of one’s inherent interest in research, i.e. the inner drive. Together, they form one’s personal research agenda.

Technical support and research funding are two other oft-cited factors that influence research participation [14]. Bakken et al. noted that a supportive environment such as mentoring, training, assistance with institutional review board approval and availability of research network were facilitators contributing to research participation [14]. We also noted that a favourable research environment facilitates research participation, but it was not the key to research participation.

Our findings have two implications for promoting primary care research. First, measures taken without considering the fundamental social environment might be futile. For example, any new or innovative measures for GPs’ research participation need to facilitate their adjustment to the GPs’ social environment. These would include taking into consideration their practice revenue, workload, organisational culture and rewards. Participation in research should be balanced with expected increased responsibilities. Second, encouraging all GPs to undertake research may not be possible because of their personal research agendas and priorities. A better approach is to identify GPs who are ‘research-ready’ and support them. GPs who are ready for research participation are those who place a high priority on research within their social environment. The methods of identifying such GPs would be an interesting and important subject of future research.

### Strengths and limitations

We drew on the strength of grounded theory methods to develop a model to understand the decision-making process of full-time practicing GPs on whether to participate in research activities. We identified a critical step in the model, fitting research agenda into the social environment, which is the core category [21,22] in the model.

We foresee different levels of research participation depending on the GPs’ research motives and environmental factors. Thus, to maximize the external validity of our findings, we involved a wide range of GPs in the study. We intentionally selected GPs with different levels of research participation, i.e. from GPs who only participated in data collection to GPs who had full involvement in a research project, to explore the interaction between the degree of interest and influence of the factors on their decision-making. We also selected GPs who had conducted research in the most challenging situation of private solo practice and GPs who worked in facilities that support research, such as those that collaborate closely with the Malaysian Ministry of Health Clinical Research Centre. We also included full-time practicing GPs in our analysis. The analytical rigor was strengthened by comparing the findings of the detailed analysis of line-by-line coding with a bird’s eye view of the transcripts provided by the research team members, who only read the transcripts without coding them. All research team members conducted at least one interview. Subsequent categorizing, rearrangement of concepts and theoretical coding were constructed through many discussions and debates among research team members. The other strength of our study was the ongoing participant recruitment and data collection after initial model construction. This theoretically driven sampling allowed greater depth and quicker saturation of our model [21]. Nevertheless, the ultimate test of the model’s external validity would be to observe the outcomes following intervention based on our model.

The analysis was based on narratives from the participants, and at best, our findings represent the reconstructed actions and mental processes of decision-making. We considered this the closest possible reflection we could achieve of the actual situation, as observation of the GPs’ actions was not feasible. We also did not present our findings to the participants or stakeholders for checking and validation. The latter step could provide further rigor to our findings. However, we do not believe this would alter the core category because our core category remained relevant and has sufficient explanatory power on the data we collected from the last few interviews. Lastly, although the model was constructed from data involving only full-time practicing GPs, we postulate that it may be relevant to academic GPs as well. While variations among different groups of professions may exist, they are likely about the factors more significant than others when deciding to participate in research. Nevertheless, future studies may be needed to confirm our postulation.

## Conclusion

We demonstrate the dynamic interplay between personal factors, social environment and organisational factors in GPs deciding to participate in research, which, to our best knowledge, the previous literature has not highlighted. Also, fitting research in their clinical practice requires active efforts from GPs to adjust their social environment in order to accommodate research activities. The degree of effort invested is directly linked to the degree of interest in research and the prioritization of the research agenda over other social environment factors. Strategies that help GPs increase their confidence in research and strengthen their research skills would encourage research participation, but have to be coupled with a supportive social environment. Thus, a more efficient measure would be to effectively identify, train and support GPs who are ready to participate in research activities, having taken into consideration their social environment. Otherwise, incentives and rewards should be established to improve the social environment.

## Supporting information

S1 File Code list for research participation project. (XLSX)
